# The association between health anxiety, physical disease and cardiovascular risk factors in the general population – a cross-sectional analysis from the Tromsø study: Tromsø 7

**DOI:** 10.1186/s12875-022-01749-0

**Published:** 2022-06-02

**Authors:** Anja Davis Norbye, Birgit Abelsen, Olav Helge Førde, Unni Ringberg

**Affiliations:** Department of Community Medicine, UiT the Arctic University of Norway, Postbox 6050, Langnes, 9037 Tromsø, Norway

**Keywords:** Health anxiety, Whiteley index, Epidemiology, Chronic diseases, Cardiovascular risk factors

## Abstract

**Background:**

Health anxiety (HA) is defined as a worry of disease. An association between HA and mental illness has been reported, but few have looked at the association between HA and physical disease.

**Objective:**

To examine the association between HA and number of diseases, different disease categories and cardiovascular risk factors in a large sample of the general population.

**Methods:**

This study used cross-sectional data from 18,432 participants aged 40 years or older in the seventh survey of the Tromsø study. HA was measured using a revised version of the Whiteley Index-6 (WI-6-R). Participants reported previous and current status regarding a variety of different diseases. We performed exponential regression analyses looking at the independent variables 1) number of diseases, 2) disease category (cancer, cardiovascular disease, diabetes or kidney disease, respiratory disease, rheumatism, and migraine), and 3) cardiovascular risk factors (high blood pressure or use of cholesterol- or blood pressure lowering medication).

**Results:**

Compared to the healthy reference group, number of diseases, different disease categories, and cardiovascular risk factors were consistently associated with higher HA scores. Most previous diseases were also significantly associated with increased HA score. People with current cancer, cardiovascular disease, and diabetes or kidney disease had the highest HA scores, being 109, 50, and 60% higher than the reference group, respectively.

**Conclusion:**

In our general adult population, we found consistent associations between HA, as a continuous measure, and physical disease, all disease categories measured and cardiovascular risk factors.

**Supplementary Information:**

The online version contains supplementary material available at 10.1186/s12875-022-01749-0.

## Background

Health anxiety (HA) is defined as a worry of disease ranging from mild worry to excessive anxiety [1–3], although previous research has most commonly employed cut-offs to define high HA. HA is associated with both increased healthcare use [4] and disability benefits [5]. In a general population, people with a history of HA are substantially more likely to experience at least one physical or mental health disorder [6]. The prevalence of people living with a physical disease is growing due to an aging population and improvements in diagnostics and treatment [7, 8]. Further, primary prophylactic treatment of cardiovascular risk factors is increasing due to routine screening [9] and a decline in the cut-off used to define people at risk. Although screening for cardiovascular risk factors has not led to an increase in mental distress [10], we have little knowledge about the association with HA. Therefore, the association between HA and physical disease and cardiovascular risk factors deserves increased attention and relevance in clinical work.

The association between HA and physical disease has mostly been explored within specific patient groups. High HA has been reported in several patient populations with different physical diseases, such as cancer [11], cardiovascular disease [12, 13], diabetes [14], and kidney disease [15]. In addition, different studies have examined disease-specific anxiety such as fear of cancer recurrence [16], fear of hypoglycaemia [17], and cardiac anxiety [18]. However, a recent review [19] proposed that these are dimensions of the broader HA concept, and pointed out that disease-specific measurements in disease-specific populations make comparison between different diseases difficult.

The association between HA and physical disease and risk factors for disease has been less explored in the general population; only three studies on the topic have been published, with inconsistent results [6, 20, 21]. To our knowledge, only one study, published by Noyes and colleagues in 2000, has examined the association between HA and various diseases in a general adult (aged 40-65 years) population [22]. They found that high blood pressure, stroke, and chronic lung disease were associated with high HA. All of these studies used a single cut-off to dichotomise high and low HA, and to-date, no one has looked at this association while measuring HA as a continuous construct. HA is reported to be unequally distributed in the population [2], with no clear cut-offs to define high HA. In accordance with Rachman [1] and Ferguson [3], we support the idea that HA in the general population should be assessed as a continuous construct.

The aim of the present paper was to examine the association between HA and 1) number of diseases, 2) different disease categories, and 3) cardiovascular risk factors in a large sample of the general population.

## Methods

### Study design and population

The Tromsø study is a large Norwegian population-based health survey, where inhabitants of the municipality of Tromsø have been invited to seven different surveys (Tromsø 1-7) since in 1974 [23]. The present paper used cross-sectional, self-reported data from Tromsø 7, which was conducted in 2015-2016. All inhabitants aged 40 years or older (*n* = 32,591) were invited by post and received two reminders to participate. Informed consent was given upon attendance, where both self-reported and clinical measures were collected. This study only utilized self-reported measurements. Of the invited participants to the Tromsø 7, 21,083 gave informed consent and participated in this study (response rate of 65%). Only information concerning age and gender of non-participants were collected.

### Variables

#### Dependent variable

We measured HA using a validated and modified one-factor, six-item Whiteley Index-6 (WI-6-R) (Table 1), which was included in the Tromsø 7 questionnaire. The WI-6-R has satisfactory psychometric properties [24] in a general population. Respondents answered each item on a 5-point Likert scale (0=“not at all”, 1=“to some extent”, 2 = “moderately”, 3=“to a considerable extent”, 4=“to a great extent”). Item scores were then summed to create a HA score ranging from 0 to 24, with higher scores indicating higher HA.

**Table 1:** Questions included in the Whiteley Index-6-R

Item	Text
1	Do you think there is something seriously wrong with your body?
2	Do you worry a lot about your health?
3	Is it hard for you to believe the doctor when he tells you there is nothing to worry about?
4	Do you often worry about the possibility that you have a serious illness?
5	If a disease is brought to your attention (e.g., via TV, radio, internet, newspapers or someone you know), do you worry about getting it yourself?
6	Do you have recurring thoughts about being ill that are difficult to get off your mind?

#### Independent variables

Participants gave information on the following diseases: heart failure, atrial fibrillation, angina pectoris, myocardial infarction, stroke, diabetes, kidney disease, chronic bronchitis/emphysema/chronic obstructive pulmonary disease, asthma, cancer, rheumatoid arthritis, osteoarthritis, and/or migraine. Response options were “no”, “yes, now”, or “previously, not now” for each disease except myocardial infarction and stroke, where only “no” and “previously, not now” were possible. Participants also reported cardiovascular risk factors (high blood pressure, use of blood pressure lowering medication, or use of cholesterol lowering medication), now or previously.

When examining the association between HA score and number of diseases (number of diseases analysis), participants were categorised according to number of diseases (0, 1, 2, 3, > 4), past or current, and cardiovascular risk factors were not counted as diseases. When examining the association between HA score and disease category (disease category analysis), we grouped the different diseases into eight disease categories, and cardiovascular risk factors were included as a separate category (Table 2).

**Table 2:** Overview of disease categories and respective included diseases from the Tromsø study: Tromsø 7 (2015-2016)

Disease category	Included diseases
No disease	None of the below mentioned
Cancer	Cancer
Cardiovascular disease	Heart failure, atrial fibrillation, angina pectoris, myocardial infarction, stroke
Diabetes or kidney disease	Diabetes, kidney disease
Respiratory disease	Asthma, chronic bronchitis/emphysema/chronic obstructive pulmonary disease
Rheumatism	Rheumatoid arthritis, osteoarthritis
Migraine	Migraine
Cardiovascular risk factors	High blood pressure, use of blood pressure or cholesterol lowering medication

#### Confounders

We included four groups of possible confounders in the analyses: disease-related variables, socioeconomic, social network, and demographic variables, all of which were taken from the Tromsø 7 questionnaire. The disease-related variables included disease in first-degree relatives and self-reported mental illness by the Hospital Anxiety and Depression Scale (HADS). Participants were asked if their first-degree relatives had any of the following: angina pectoris, stroke, asthma, diabetes, breast cancer, prostate cancer, colon cancer, or myocardial infarction before the age of 60. Participants were categorised as “yes” if they reported that their first-degree relatives had one or more of these diseases, and “no” if they had none of them. Disease in first-degree relatives was chosen as a confounder as we hypothesised that HA may be affected by disease in close family [1], and since many of the diseases can be hereditary. Mental illness is associated with HA [6] and physical disease [25–27]. We therefore included the measurement tool HADS [28] as a confounder. HADS is a questionnaire based on participants’ responses to 14 questions concerning symptoms of anxiety and depression in the last week, with a total range of 0-42. Due to the diverse use of cut-offs for HADS total score [29], we used HADS as a continuous measure, except for descriptive purposes.

Socioeconomic variables were considered confounders based on associations with both HA [2] and physical disease [30]. Participants reported highest level of completed education (primary education up to 10 years of schooling, vocational/upper secondary education ≥3 years, college/university < 4 years, or college/university ≥4 years) and annual household income, which was categorised as low (NOK < 451,000), lower middle (NOK 451-750,000), upper middle (NOK 751000-1 million), or high (NOK > 1 million). There were two social network variables: participation in organised activities and friendship. Both are associated with HA [2] and physical disease [31]. Response options for participation in organised activities were “never or just a few times a year”, “1-2 times a month”, “approximately once a week”, or “more than once a week”. The friendship variables included two questions: “Do you have enough friends who can give you help and support when you need it?” and “Do you have enough friends with whom you can talk confidentially?” Response options were “yes” and “no”, and these were merged and coded as “no”, for those who answered “no” to both questions; “to some extent”, for those who answered “yes” to only one question; and “yes”, for those who answered “yes” to both questions. Finally, demographic variables included gender and age as of 31 December 2015.

### Statistical analyses

No participants were excluded prior to the analyses, but missing values were consequently excluded in the analyses and all results are therefore presented as complete-case. In the disease category analysis, disease categories were exclusive, thus participants with diseases in two different categories (e.g. cancer and angina pectoris) were excluded. However, participants were not excluded if they had cardiovascular risk factors in addition to a specific disease category, e.g. high blood pressure in addition to cancer. Participants could state several diseases within each disease category, e.g. angina pectoris and heart failure. If they answered “previously, not now” for one disease and “yes, now” for another within the same disease category, they were categorised as “yes, now”. We set the reference group for all analyses as participants who reported both no current or previous physical disease and no cardiovascular risk factors (healthy reference group).

In the descriptive analyses, frequency distributions are presented for categorical variables, and mean (Standard deviation, SD) median [quartiles 1, 3] for continuous variables. All analyses were performed with STATA version 16.1 (STATA Corp LP, College Station, Texas, USA).

Due to the non-normal and highly skewed distribution of the dependent variable HA, we used bivariate and multivariate exponential regression analyses to detect associations. The regression coefficients in the estimated models are presented with the exponentiated beta [exp(b)], where exp.(b) describes the percentage change in the WI-6-R score relative to the reference category for the different other categories.

The unadjusted regression model included the disease category independent variable, and the adjusted model adjusted for all specified confounders. We tested for two possible interactions: physical diseases and education and physical diseases and age, with the hypotheses that people with a higher education level would have more resources to handle disease, and that younger and older participants would deal with illness differently. However, no interactions were evident.

### Ethics

The study was conducted in accordance with the Declaration of Helsinki and was approved by the Regional Committee for Medical and Health Research Ethics (REC North) in Norway (ID 2016/1793). All participants gave written informed consent before admission.

## Results

### Participant characteristics

Of the 21,083 Tromsø 7 participants (age range: 40-99 years; mean 56, SD 11), 52.5% were women. Supplementary Table 1 shows participant characteristics of the confounders. In total, 18,432 participants had complete information on the number of diseases and cardiovascular risk factors. Of these, 17,997 had completed the WI-6-R. The mean (SD) median [quartiles] HA score was 3.26 (3.39), 2[1,5] out of 24 points in the population, and HA scores increased with increasing number of diseases (Table 3).

**Table 3:** Mean (SD) and median [quartiles 1, 3] health anxiety (HA) score according to number of physical diseases, disease category, and cardiovascular risk factors. Data from The Tromsø study: Tromsø 7 (2015-2016)

				Mean (SD), median [quartiles] HA score as indicated by the Whiteley Index-6-R
		N	Percent	
Number of physical diseases, 5 categories	None	7231	43%	2.28 (2.83), 1 [0-4]
	One disease	5801	35%	2.92 (3.14), 2 [0-4]
	Two diseases	2342	14%	3.54 (3.53), 3 [1-5]
	Three diseases	818	5%	4.23 (3.73), 3 [1-6]
	Four or more diseases	389	2%	4.80 (4.24), 4 [2-7]
	Total	16,581		
Disease category				
Cancer	No	7231	93%	2.28 (2.83), 1 [0-4]
	Previously, not now	450	6%	2.84 (3.14), 2 [0-4]
	Yes, now	127	2%	4.59 (4.09), 4 [1-6]
	Total	7808		
Cardiovascular disease	No	7231	88%	2.28 (2.83), 1 [0-4]
	Previously, not now	694	8%	2.95 (3.05), 2 [0-5]
	Yes, now	295	4%	3.51 (3.66), 2 [1-5]
	Total	8220		
Diabetes or kidney disease	No	7231	93%	2.28 (2.83), 1 [0-4]
	Previously, not now	154	2%	2.70 (2.76), 2 [1-4]
	Yes, now	371	5%	3.65 (3.62), 3 [1-5]
	Total	7756		
Respiratory disease	No	7231	87%	2.28 (2.83), 1 [0-4]
	Previously, not now	355	4%	2.66 (2.72), 2 [0-4]
	Yes, now	724	9%	3.02 (3.28), 2 [1-4]
	Total	8310		
Rheumatism	No	7231	81%	2.28 (2.83), 1 [0-4]
	Previously, not now	130	1%	2.12 (2.90), 1 [0-4]
	Yes, now	1620	18%	2.97 (3.15), 2 [1-5]
	Total	8981		
Migraine	No	7231	84%	2.28 (2.83), 1 [0-4]
	Previously, not now	629	7%	2.67 (2.84), 2 [0-4]
	Yes, now	779	9%	2.86 (3.17), 2 [0-5]
	Total	8639		
Cardiovascular risk factors	No	7231	78%	2.28 (2.83), 1 [0-4]
	Yes	2096	22%	2.65 (2.86), 2 [0-4]
	Total	9327		

For all the investigated disease variables, having no disease was the most common, with increased HA observed among those with one or more diseases, those who fell into any disease category, and those with cardiovascular risk factors. For most diseases, the mean HA score was higher among those with current disease compared to those with previous disease.

### Association between health anxiety, physical disease, and cardiovascular risk factors

There was a significant, positive association between HA score and number of diseases, and between HA score and disease categories (Table 4). In the fully adjusted model, participants reporting one physical disease had 29% higher HA scores than the healthy reference group, and participants with four or more physical diseases had a two-fold increase in HA scores compared to the healthy reference group. HA was consistently associated with all disease categories, with higher HA scores in all disease categories compared to the healthy reference group.

**Table 4:** Association between health anxiety score and number of diseases, and between health anxiety score and disease category, presented with exponential regression coefficients. Data from The Tromsø study: Tromsø 7 (2015-2016)

		Unadjusted model		Adjusted model^a^	
		Exp(b)	95% CI	Exp(b)	95% CI
Number of diseases, 5 categories	None	1		1	
Unadjusted model, *N* = 16,169 Adjusted model, *N* = 13,971	One disease Two diseases Three diseases Four or more diseases	1.28^c^ 1.55^c^ 1.85^c^ 2.11^c^	1.23 – 1.32 1.48 – 1.63 1.72 – 2.00 1.90 – 2.34	1.29^c^ 1.53^c^ 1.89^c^ 2.09^c^	1.24 – 1.34 1.45 – 1.61 1.74 – 2.05 1.85 – 2.36
Disease category					
Cancer	No	1		1	
Unadjusted model, *N* = 7655	Previously, not now	1.24^c^	1.13 – 1.37	1.32^c^	1.18 – 1.46
Adjusted model, *N* = 6761	Yes, now	2.01^c^	1.68 – 2.41	2.19^c^	1.80 – 2.69
Cardiovascular disease	No	1		1	
Unadjusted model, *N* = 8047	Previously, not now	1.30^c^	1.19 – 1.43	1.29^c^	1.17 – 1.40
Adjusted model, *N* = 7076	Yes, now	1.44^c^	1.31 – 1.59	1.50^c^	1.31 – 1.72
Diabetes or kidney disease	No	1		1	
Unadjusted model, *N* = 7597	Previously, not now	1.18^b^	1.01 – 1.39	1.13	0.95 – 1.34
Adjusted model, *N* = 6702	Yes, now	1.60^c^	1.43 – 1.78	1.60^c^	1.42 - 1.81
Respiratory disease	No	1		1	
Unadjusted model, *N* = 8149	Previously, not now	1.17^b^	1.05 – 1.30	1.13^b^	1.01 – 1.26
Adjusted model, *N* = 7208	Yes, now	1.32^c^	1.23 – 1.43	1.36^c^	1.25 – 1.48
Rheumatism	No	1		1	
Unadjusted model, *N* = 8776	Previously, not now	0.93	0.78 – 1.11	1.05	0.87 – 1.27
Adjusted model, *N* = 7694	Yes, now	1.30^c^	1.23 – 1.38	1.38^c^	1.29 – 1.47
Migraine	No	1		1	
Unadjusted model, *N* = 8478	Previously, not now	1.17^c^	1.08 – 1.27	1.12^b^	1.03 – 1.21
Adjusted model, *N* = 7551	Yes, now	1.26^c^	1.17 – 1.35	1.13^c^	1.03 – 1.23
Cardiovascular risk factors	No	1		1	
Unadjusted model, *N* = 9132	Yes	1.16^c^	1.10 – 1.23	1.24^c^	1.17 – 1.31
Adjusted model, *N* = 8014					

^a^Confounders included: age, sex, education, household income, disease in first-degree relatives, HADS score, friendship, and participation in organised activity. ^b^ Significant below 0.05 level. ^c^ Significant below 0.01 level. *CI* Confidence interval

For all disease categories, current disease was associated with higher HA scores than previous disease. Moreover, in most disease categories except previous diabetes or kidney disease and previous rheumatism, those with previous disease had higher HA scores than the healthy reference group. Participants with current cancer had the highest HA scores; twice as high as in the healthy reference group. Participants with current cardiovascular disease and current diabetes or kidney disease had an increase in HA scores of 50 and 60%, respectively, compared to the healthy reference group. Participants with cardiovascular risk factors also had a significant, 24% increase in HA scores compared to the healthy reference group.

## Discussion

The aim of our study was to explore the association between HA and physical disease. We found several important and consistent results: Increasing number of diseases was associated with significantly higher HA scores. Both people reporting current and previous disease had higher HA scores compared to the healthy reference group. Cancer, cardiovascular diseases, and diabetes or kidney disease showed the strongest association with HA. Finally, participants with cardiovascular risk factors had significantly higher HA scores than the healthy reference group. To our knowledge, this is the first paper to demonstrate how HA is associated with both number of physical diseases, different disease categories, current and previous disease, and cardiovascular risk factors in the general population.

The HA scores we observed among those with four or more diseases were twice as high as scores among those with no diseases, and we believe this to be a novel finding. Although some studies have found an association between high HA and having a disease [6, 20], only one previous study has examined the association between HA and the number of physical diseases [21]. In contrast to our study, they did not find any significant association between HA and increasing number of diseases. However, they used a cut-off to dichotomise high and low HA, which might have obscured a significant trend. Unlike previous studies that used different cut-offs to measure HA [6, 21, 22], we utilised HA as a continuum, which may better represent the phenomenon of HA.

As this is a cross-sectional study, it cannot determine causality. We speculate that the observed association may be explained by the presence of disease increasing the risk of having higher HA score [19]. However, high HA is also associated with high healthcare use [4], which may increase the probability of acquiring a diagnosis. In addition, we do not know if HA is itself is a risk factor for future disease. High levels of HA has been found associated with increased risk for ischaemic heart disease [32], whereas Knudsen and colleagues [33] found that high HA was associated with increased cancer incidence in men. Further, no association was found between HA and cancer incidence in a cohort of women, but high HA was associated with increased all-cause mortality [34]. To better examine and understand causal directionality in the relationship between HA and different diseases, and to investigate if gender influences the role of HA, a cohort study design is warranted.

### The association between HA and different diseases

We found significant associations between HA scores and all disease categories investigated in this study, which included the most common non-communicable chronic diseases. Our results are in accordance with previous findings of high HA in patient populations [11, 14, 15, 18, 35]. Current cancer, cardiovascular diseases, and diabetes or kidney disease was associated with the highest HA scores. Fear of cancer and cardiovascular disease is common in people with HA [1, 36]. Having current diabetes or kidney disease was also highly associated with HA scores in this study. Diabetes control requires strict adherence and bodily monitoring. Fear of complications was strongly associated with HA in a previous population of patients with diabetes [14], and may explain the high association between HA and this disease category in our study. Assuming that the diseases occurred prior to the HA, it could be reasonable to suggest that the bodily monitoring and fear of the fatal outcome may explain the high associations in this general population.

Another consistent finding was that those reporting previous disease had lower HA scores than those with current disease, but they had still higher scores than the healthy reference group. Although most of the diseases included in our study are considered chronic, their symptoms can be reduced by proper treatment. We therefore speculate that some of our participants may have some disease, but proper management of that disease decreased both their symptom burden and HA.

Interestingly, we found a significant association between HA and cardiovascular risk factors, with a coefficient similar to coefficients for migraine and respiratory disease. The impact of a 24% increase in HA score in otherwise healthy persons indicates a potential health burden on a population level. In Norway, the proportion of 70-74-year-olds taking blood pressure- or cholesterol lowering medication is increasing [37]; and was as high as 57% in 2016 [38]. Primary healthcare in Norway is well-functioning [39]. It is reasonable to assume that those who report a cardiovascular risk factor receive treatment, and thereby are at lowered risk for future cardiovascular disease. It is therefore interesting that we observed such a pronounced association between cardiovascular risk factors and HA. This significant association is important in the discussion of adverse effects in identifying people “at risk”.

### Possible cohort effect

Older age is associated with lower HA [40], and as physical diseases are more prevalent in older individuals, we hypothesised that the association between HA and disease may differ by age. However, we did not find any significant interaction, indicating that having a disease is not associated with higher HA in younger (40 years) compared to older age groups. Moreover, mean HA has increased in student populations in the past three decades [41], and if there is, in fact, a cohort effect, it is likely that today’s youth may experience an even higher HA later in life due to the increased prevalence of disease in older age groups.

### Methodological considerations

As this study uses a cross-sectional study design, we cannot determine whether HA occurs prior to the disease or in response to the disease, and caution should be taken when making assumptions of the directions of associations. Nevertheless, we believe that this study shows novel findings of associations in a general population, which may lay the foundation for future prospective studies.

A strength of this study is the large, representative sample from the general population, which enabled us to examine the association between HA and different diseases. We chose to use a validated measurement tool, which is a strength in the research field of HA, and used a revised version that distinguished the cognitive construct of illness worry from the presence of physical symptoms [24]. Comparisons between studies are difficult due to the use of different HA measurement tools [19] as well as reporting of different diseases [7]. Although our results align with studies in other countries [6, 22] and patient populations [11, 12, 14, 15], our sample is exclusively from inhabitants in a specific geographic region in Norway, and replication in other populations would allow for further generalisation of the results.

All our data on the occurrence of disease was self-reported, and any misclassification may be due to recall bias. If the reporting of disease is related to HA, e.g. if those with low HA under-report disease more than those with higher HA, this could bias our results. However, a Norwegian study examining consistency among self-reported diagnoses and clinical registries found good overall consistency [42].

In our study, we asked about current or previous disease, not duration of disease. One article examining HA in cancer patients found that HA was consistent over time after diagnosis and also during remission [43], and high HA has also been described as stable over time [44]. However, one study carried out in a sample of patients with diabetes found that high HA was most highly associated with a recent diagnosis [14]. Another factor concerning morbidity is severity of disease (risk of fatal outcome, the need for disease monitoring, chronic disability, etc.), as most of the diseases in our disease categories may have a wide range of severity. Interestingly, Tu et al. [15] found that increased HA was independent of kidney disease severity. However, as disease severity and duration may have influenced participants’ responses, the lack of this information may increase any heterogeneity of the associations presented.

The introduction to the questionnaire, stating the timeframe of the past 12 months, was omitted in the survey. This limits our knowledge of the timeframe during which the participants answered. Although severe HA has been found to be stable over time [44], this is unknown for people with lower HA scores.

As in all survey research, selection bias may occur. Unfortunately, we have no information on factors related to non-response in the Tromsø 7, other than age and gender. However, a similar survey found that chronic diseases, e.g. diabetes, was related to non-attendance [42], indicating that survey populations may be healthier than non-respondents. Although not previously examined, it has been hypothesised that, in contrast to other mental illnesses, people with HA attend studies that are advertised as a “health check-up” [5], which was done in Tromsø 7. If the participants in the Tromsø 7 were healthier, whilst having higher HA, our results may be biased towards the null.

As Lebel et al. [19] pointed out, there is an overlap between disease-specific measures and HA. Although disease-specific HA may be more precise than the more general concept of HA, we believe that HA should be used in a larger and comparative perspective.

### Clinical implications

Our study demonstrates a consistent trend in the association between HA and physical disease which confirms knowledge from clinical practice and highlights the importance of assessing and addressing HA in patients with either current or previous disease. Past research has shown associations between HA and a wide range of diseases in patient populations. In line with those results, we suggest that while the proportion of HA may not vary considerably between diseases, the mere presence of disease is associated with higher HA. This association is relevant from a clinical perspective, as over 50% of our study sample had one or more diseases (Table 3). Severe HA is associated with a wide range of negative consequences, such as functional impairment, activity limitations, psychological distress [6], and increased healthcare use and work disability [4, 5, 45], and should be managed through targeted treatment to reduce associated negative consequences. However, as we have found in this study, increasing number of diseases is associated with higher HA, but overall, HA remains low. However, some studies found an association between lower HA score and higher healthcare use [46, 47] and therefore we do not know how low HA is relevant from a clinical perspective. From a healthcare systems perspective, it is important to account for HA in the management of disease, particularly in those with increased number of physical diseases. Even when HA is not severe enough to require diagnosis and targeted treatment, we believe it important that healthcare personnel acknowledge and address the additional burden that HA may place on persons with current or previous physical disease and those with cardiovascular risk factors.

## Conclusion

In our general adult population, we found consistent associations between HA and physical disease and cardiovascular risk factors. The highest HA scores were found among those with four or more diseases and participants with current cancer, but the positive association was consistent in all disease categories and cardiovascular risk factors. Previous disease was also associated with increased HA. Our results indicate that HA should merit closer attention in future research on populations with physical disease and risk factors for disease.

## Supplementary Information

**Additional file 1: Supplementary Table 1.** Population characteristics of participants in the Tromsø study: Tromsø 7 (2015-2016) by confounding variables.

## Data Availability

The data that support the findings of this study are available from the Tromsø study but restrictions apply to the availability of these data, which were used under license for the current study, and so are not publicly available. Data are however available from the authors upon reasonable request and with permission of the Tromsø study: https://uit.no/research/tromsoundersokelsen

## Abbreviations

HA: Health Anxiety
WI-6-R: Whiteley Index 6-R
HADS: Hospital Anxiety and Depression Scale
