# Supplementary material for: The association between health anxiety, physical disease and cardiovascular risk factors in the general population – a cross-sectional analysis from the Tromsø study: Tromsø 7
Source: BMC Prim Care. 2022 Jun 2;23:140. doi: 10.1186/s12875-022-01749-0 (PMC9161473; doi:10.1186/s12875-022-01749-0)
Supplement: Supplementary file 1 — Additional file 1: Supplementary Table 1. Population characteristics of participants in the Tromsø study: Tromsø 7 (2015-2016) by confounding variables. [file 12875_2022_1749_MOESM1_ESM.docx]

**Supplementary table 1**. Population characteristics by confounding variables

| Variable |  |  | |
| --- | --- | --- | --- |
|  | Categories | N | Percent |
| Age | 40-49 years  50-59 years  60-69 years  70-79 years  80 years or older | 6 432  6 035  5 179  2 676  761 | 31 %  29 %  25 %  13 %  4 % |
|  | Total | 21 083 |  |
| Gender | Female  Male | 11 074  10 009 | 53 %  47 % |
|  | Total | 21 083 |  |
| Educational level | Primary/partial secondary education  Upper secondary education  Tertiary education, short  Tertiary education, long | 4 796  5 756  4 008  6 145 | 23 %  28 %  19 %  30 % |
|  | Total | 20 705 |  |
| Household income | Low (less than NOK 451 000)  Lower middle (NOK 451-750 000)  Upper middle (NOK 751 000-1 million)  High (More than NOK 1 million) | 4 545  5 884  4 741  5 015 | 23 %  29 %  23 %  25 % |
|  | Total | 20 185 |  |
| Disease in first-degree relatives | No  Yes | 15 894  4 505 | 78%  22 % |
|  | Total | 19 892 |  |
| HADS total score | Below 11 points  11 points or more | 15 895  3 237 | 83 %  17 % |
|  | Total | 19 132 |  |
| Friendship | No  To some extent  Yes | 1 621  1 774  17 117 | 8 %  9 %  83 % |
|  | Total | 20 512 |  |
| Organized activity | Never or just a few times a year  1-2 times a month  Approximately once a week  More than once a week | 11 310  4 981  2 587  1 856 | 55 %  24 %  13 %  9 % |
|  | Total | 20 744 |  |
